# Consciousness: its goals, its functions and the emergence of a new category of selection

**DOI:** 10.1098/rstb.2024.0310

**Published:** 2025-11-13

**Authors:** Eva Jablonka, Simona Ginsburg

**Affiliations:** ^1^ Department of Humanities, Tel Aviv University, Tel Aviv-Yafo, Tel Aviv District, Israel; ^2^ London School of Economics and Political Science, London, UK; ^3^ Department of Natural Science, The Open University of Israel, Raanana, Israel

**Keywords:** Cambrian, choice, mental, consciousness-guided selection, signalling, social selection, unlimited associative learning (UAL)

## Abstract

We suggest that the emergence of consciousness in living organisms entailed new goals and new functions, which gave rise to a new category of selection, which we call mental selection. Mental selection involves ontogenetic choices that are directed towards consciously perceived and affectively evaluated patterns. It expands the types, targets and regimes of natural and sexual-social selection and is a scaffold on which human artificial selection emerged. We suggest that the functional effects of consciousness and the mental selection which it affords, were driven and enabled by the evolution of an open-ended form of associative learning (unlimited associative learning (UAL)). UAL enables animals to discriminate between composite percepts and acts and permits plastic self-learning and goal-directed behaviour driven by flexibly prioritized physiological needs, which enable flexible adjustments to a huge range of conditions and events during the animal’s lifetime. We propose that UAL-based signal selection, involving for example, predator–prey, sexual and other social interactions, led to the evolution of intricate perceptual, emotional and motor patterns that could not have existed before consciousness evolved. These patterns, which can be thought of as signatures of consciousness, first appeared in the Cambrian era and scaffolded the evolution of imaginative animals and reflective humans.

This article is part of the theme issue ‘Evolutionary functions of consciousness’.

## 1. Introduction

Charles Darwin’s theory of evolution by natural selection is one of the most powerful unifying theories of modern science. When recursively applied it has an explanatory power, which Daniel Dennett analogized to ‘universal acid’, dissolving supposedly unresolvable problems and offering a scientific explanation to the myriads of goal-directed actions and functional patterns in the living world [1]. The notions of biological goal, value, function and adaptation are therefore intimately related to that of selection and form a theoretical nexus.^1^

Since it was put forward and developed by Darwin, different types and manifestations of selection were explored. Darwin [2] distinguished between natural selection (for survival and reproduction), intrasexual and intersexual selection (selection for reproductive success through mate attainment, which may conflict with selection for survival), and artificial selection by human intelligent design. Darwin also considered different targets of selection such as individuals, families and groups and discussed the efficacy of selection and the environmental regimes that favoured selection at these levels of organization [2,3]. Herbert Spencer further extended the notion of selection onto the social and cultural realms [4], and August Weismann and Wilhelm Roux applied it to the sub-individual level, to developmental selection among parts of the body and selection among hereditary units within cells [5,6]. Although most notions of selection involved selection among reproducing units (such as cells, organisms, families, groups) it was recognized that the notion of selection is broader than classical Darwinian selection among reproducing entities and may involve differential persistence or ‘lastingness’, as in Roux’s struggle of parts in the body during development.

During the twentieth century many *regimes of selection* were studied both theoretically and empirically. Directional selection, frequency-dependent selection, density-dependent selection, stabilizing selection, canalizing selection, diversifying selection, sexual selection, K- and r-selection are among the regimes of selection explored; so were *different levels and targets of selection* such as individual, family and social and cultural group selection, ontogenetic selection among somatic cells and germ cells within the organism, selection at the gene level (e.g. through meiotic drive or through transposable elements’ differential replicative amplification), selective stabilization of interactions of cells or cell groups within the organisms such as neuronal cells and cell-groups [7,8], and selection for persistence among chemical networks that preceded the emergence of life on earth [9]. A general notion of selection, which included selection among reproducing units as well as selection of a sample from a set (sample selection) was discussed by eminent philosophers of biology (e.g. [1,10]) and clearly articulated by George Price, who developed the most general formalization of selection, the now famous Price equation. ([11]; for a more recent discussion of sample selection and classical Darwinian selection see [12]). An even more general theory of selection focuses on signatures of selection and combines the minimal number of steps required for the assembly of a composite entity with the number of such entities, thus capturing the amount of memory necessary to produce the selected, historically contingent, composite [13].

A review of the immense literature on the notion of selection, its classification and the interactions among different forms and levels of selection between and within organisms, among organismal products and among groups is beyond the scope of an article. However, there is a curious oversight that is shared by most scholars in the field. The general implicit assumption regarding selection in evolutionary biology is that (with the exception of humans) there is no difference—neither in terms of outcomes nor dynamics—between selection mediated by conscious non-human organisms and selection mediated by non-conscious ones. Although most evolutionary biologists agree that all living organisms are active agents and that their activities affect their evolutionary trajectories, mental agency is not assumed to entail specifically evolved functions and selection modes that are different from those entailed by non-mental agency. Indeed, mental agency and mental causation (and their evolutionary effects) are *not* part of Nikolaas Tinbergen’s suggested general frameworks of explanation in biology, they are not what he called ‘causes’ [14]. Tinbergen’s causes include phylogenetic ‘ultimate’ causes, functional causes which provide explanation in terms of current utility, developmental causes that give an account in terms of ontogenetic construction of the trait of interest, and immediate causes—an account in terms of currently operating underlying mechanisms. Tinbergen regarded his four causes as both necessary and sufficient for the comprehensive scientific study of all living organisms [15]. Tinbergen’s and others’ implicit conjecture has been that although function may include mental causation, such causation does not have a special theoretical status.^2^ We need not assume, according to Tinbergen, that a non-human organism such as an elephant is conscious in order to fully explain the nature of the various adaptations that it, and the species with which it interacts, display. In other words, the conscious nor non-conscious state of a non-human organism’s behaviour is assumed to be irrelevant to the explanation of the functional patterns and rates of evolutionary change in its own species and in the species (conscious or non-conscious) that interact with it.

There were a few exceptions to this view. In his book on sexual selection, Darwin [3] claimed that selection through mate choice requires mental states such as desires, an idea that was also briefly discussed in a footnote by Prum [16] as well as by Denis Noble [17] and by us [18,19]. Noble called such selection ‘intentional selection’ and included in this category of selection also some predator–prey interactions. However, neither Darwin, nor Prum or Noble pointed to theory-based arguments that specified the functions of consciousness, which led to new patterns of perceptions, actions and interactions.

We suggest that mental selection—selection that is guided by mental states and is only possible when animals are conscious—is a distinct type of natural and sexual/social selection. It requires its own category alongside classical natural selection and artificial selection because it introduces new values and goals—the satisfaction of felt needs and the evaluation of their perceived predictors—which alter the interactions among living organisms and have profound evolutionary effects.

We see the conscious mode of being as the outcome of a major teleological transition in evolution. We recognize three such transitions: the transition to a living mode of being, the transition to a living-conscious mode of being and the transition to a living-conscious-rational mode of being. Each of these transitions is characterized by new goals and values [18,20] and by the emergence of corresponding types of selection—natural selection that does not require mentality, which emerged when life first evolved; mental selection (on which we focus here), which emerged when consciousness evolved, and artificial selection by humans, which emerged with consciousness-based rationality. We believe that Darwin was right to make a conceptual distinction between natural selection and artificial selection because artificial selection has led to evolutionary patterns that would be impossible without human reflective intelligence. We argue that the category of mental selection, which points to the selection exerted by conscious, non-human animals, has also led to new evolutionary patterns and interactions that would be impossible without it, and it too should be conceptually distinguished from other forms of natural selection. We suggest that mental selection is *a special form of natural and developmental selection* requiring complex cognition (which on our theory, entails consciousness) leading to unique selected effects that are inconceivable without it. It can be considered as an intermediate category of selection—between natural selection that is not guided by mental states and human artificial selection driven by rational considerations.

The goals, functions and evolutionary effects that one attributes to consciousness and to consciousness-guided selection, depend on the theory of consciousness one holds. Here we present an evolution-based theory of consciousness (§2) and argue that according to this theory, conscious agents have distinct goals that are not available to non-conscious agents (§3). We propose that the functional capacities of these conscious agents have led to distinct, evolved sensory, motor and behavioural composite signalling patterns, as well as cognitive and affective adaptations, and that although mental selection is exerted only by some (not all) non-human animals, it has broad effects apparent in all organisms, conscious and non-conscious, that interact with them (§4). According to the theory we present, consciousness emerged during the Cambrian era, and the mental selection that consciousness entailed was one of the factors that spurred the Cambrian explosion and has been a dominant factor in the evolution of life ever since (§5). This has implications for theorizing about evolution: first, since consciousness is based on new values and leads to distinct evolutionary effects, the selection mediated by consciousness should be recognized as a distinct category of selection; second, which theory of consciousness one endorses makes a difference to theorizing about evolution (§6).

## 2. The origins of the mental: learning as the driver of the evolution of consciousness

Most biological theories of consciousness assume that consciousness is an evolved mode of being that emerged after life and the nervous systems had appeared. However, most of these theories are not centred on the evolutionary origins, history, adaptive significance and evolutionary effects of consciousness. There are some notable exceptions [18,21–29] but none of these suggests that consciousness led to the emergence of a new *category* of selection.

Before we describe our own evolution-centred theory, which focuses on the evolutionary origins of consciousness, its functions and its evolutionary effects, and explain why consciousness led to a new type of selection, we need to clarify how we use this highly polysemic term. We consider consciousness and sentience (we use these two terms as synonyms) as a mode of being that is characterized by subjective, intrinsically private phenomenal experiencing. Such experiencing includes perceiving the world and the body (e.g. seeing red, smelling jasmine, sensing touch) and feeling (e.g. pain, pleasure, joy, hunger), and, for some animals, also experiencing virtual realities (re-experiencing the past *as a past*, imagining-planning for the future). All subjective experiencing happens from a point of view and is felt to be ‘owned’ by the animal. On our approach, the most basic manifestations of consciousness do not include the experiencing of metacognitively constructed virtual realities and do not require language. Imaginative consciousness and consciousness based on symbolic representation and communication evolved later [30].

We described our approach to consciousness in detail in a book [18] and several papers including two target articles [20,31–34]. However, since the attribution of the functions and the signatures of consciousness depend on the theory of consciousness one holds, we present an outline of our theory here.^3^

### (a) The structure, dynamics and evolutionary transition marker of minimal consciousness: unlimited associative learning

**Figure 1.**
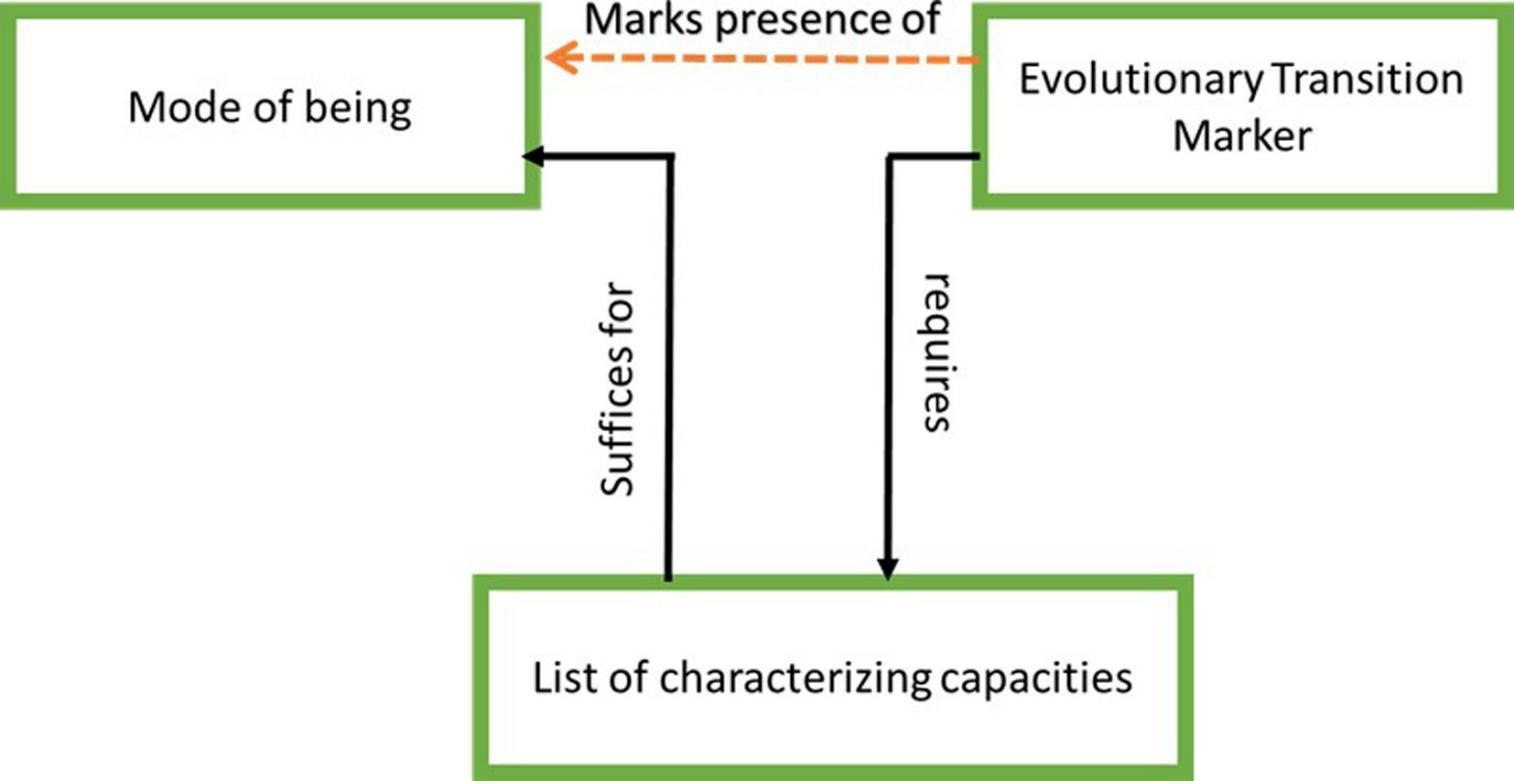
The general idea of an evolutionary transition marker (ETM). An ETM is a diagnostic capacity which requires that all the consensus properties that are jointly sufficient to attribute a particular mode of being to an entity, are in place. It therefore marks the mode of being of interest (minimal life, minimal consciousness, rational reflectiveness) [37].

In order to uncover the dynamics and structure of minimal consciousness, we used the methodology that the Hungarian chemist Tibor Gánti developed for his investigation of minimal life and the origins of life [35]. Gánti began with a compilation of characteristics of life that most biologists, chemists, geologists and philosophers (who may passionately disagree about the way that life originated) consider to be jointly sufficient for characterizing a minimal, evolutionarily persistent living system. He identified eight jointly sufficient and partially overlapping capacities of minimally living systems: maintenance of a boundary, metabolism, dynamic stability, information storage, regulation of the internal milieu, growth, reproduction and irreversible disintegration (death). On the basis of this consensus list, he constructed a model of three coupled reactions systems—a metabolic system, a membrane system and an informational-regulatory system—which, through their coupled dynamics, implement these capacities (he called his model ‘chemoton’). He also identified an experimentally tractable marker of a minimal living system: unlimited heredity—the capacity to form lineages that vary in open-ended ways from the initial system, so the number of possible different variants is vast. Any autonomous system endowed with open-ended heredity must show all the capacities in the consensus list ([35]; see [36], for elaboration). This means that if we find a system with the capacity for unlimited heredity anywhere in the universe, we should be able to re-construct or reverse-engineer on its basis the simplest autonomous (self-maintaining and self-generating) system that enables it. It is this enabling system, which he considered a minimal living system. We generalized Gánti’s notion and called the diagnostic capacity that requires that all the properties attributed to a particular teleological mode of being are in place, an evolutionary transition marker (ETM; figure 1).

Following Gánti we started our study of minimal consciousness with a list of partially overlapping characteristics, which most scholars (neurobiologists, cognitive scientists, philosophers, psychologists) see as characterizing a minimal mode of phenomenal consciousness (—the consensus list) in living organisms. This list includes:

(i) percepts’ unification and differentiation. Objects are perceived as unified totalities, yet their parts can be discerned; an apple is perceived as red, round, smooth and fragrant;
(ii) flexible value attribution: a percept or an act can have different values depending on conditions. It is good in condition A, bad in B, neutral in C; the value of different percepts can also be differently prioritized—evaluated relatively to other percepts—in different conditions;
(iii) exploration and stabilization processes, underlying vigilant and selective attention (exclusion of irrelevant information and amplification of salient information) and learning processes;
(iv) intentionality (aboutness), which entails mapping of world, body and their relationships;
(v) temporal depth: persistence and integration over time, so percepts have duration;
(vi) embodiment and agential plasticity that enables flexibly controlled voluntary motor actions;
(vii) registration of self–other distinction from a point of view and a sense of self: identical self-generated and world-generated stimuli are distinguished from an individual internal, stable perspective; and
(viii) global accessibility and broadcast, involving interactions among perception, memory, evaluations and actions representations.

An extended discussion of these attributes, and their neural, behavioural and cognitive correlates is presented in our previous publications ([18,37], [31, table 1]). Our main point is that minimal subjective experiencing is *constituted* by the coupled and evolved cognitive-neuronal processes underlying the capacities in the above list, just as life is constituted by the interactions of the complex coupled chemical reaction systems that implement the life-characteristics in Gánti’s list.

We suggested that the ETM of minimal consciousness in living organisms, which entails the capacities in the consensus list, is an open-ended, generative, domain-general, recursive and representational type of associative learning, which we called unlimited associative learning (UAL). UAL, a complex cognitive capacity, enables an organism to exhibit the following, partially overlapping, learning capacities:

(i) the organism can learn to discriminate and compare among, salient, novel (not previously encountered and learned) differently organized, multi-featured yet unified stimuli such as apples differing in colour, form, texture and smell. This requires sensory unification and differentiation, perceptual mapping and a capacity for updating the values of compound stimuli and actions from a stable point of view;
(ii) the organism can learn to attach different motivational values to the novel learned composite patterns of salient stimuli and actions depending on conditions (e.g. X is good in environment 1 bad in environment 2, neutral in environment 3). This enables, in addition to reverse learning also flexible prioritization and trade-offs among multiple stimuli and actions. The capacity for such learning points to a flexible evaluation system and to a registration of self-other distinction (which is necessary for eliciting and coordinating actions);
(iii) the organism can learn even when there is a time gap between a novel predictive stimulus and the reinforcement (trace-conditioning). Holding compound patterns in working memory points to a capacity for integration over time; and
(iv) the organism can learn to use previously learned percepts and motor actions as the basis for subsequent learning of new ones (e.g. second-order conditioning, generalization, categorization, transfer). This points to a flexible value system, a stable ‘point of view’, unification/differentiation and global accessibility.

These learning capacities are jointly necessary for attributing UAL to an animal. They imply that the animal has representations that guide goal-directed behaviours, in the sense discussed by Dickinson & Balleine [38]. They argued that goal-directed behaviour which implies consciousness requires that animals form representations of the instrumental contingency between their actions and the outcomes of these actions, as well as representations of the outcomes as goals for the agent. Patterns of learned sensory predictors of reinforced outcomes guide this intentional behaviour (see [39] for a discussion of this view of consciousness)

**Figure 2.**
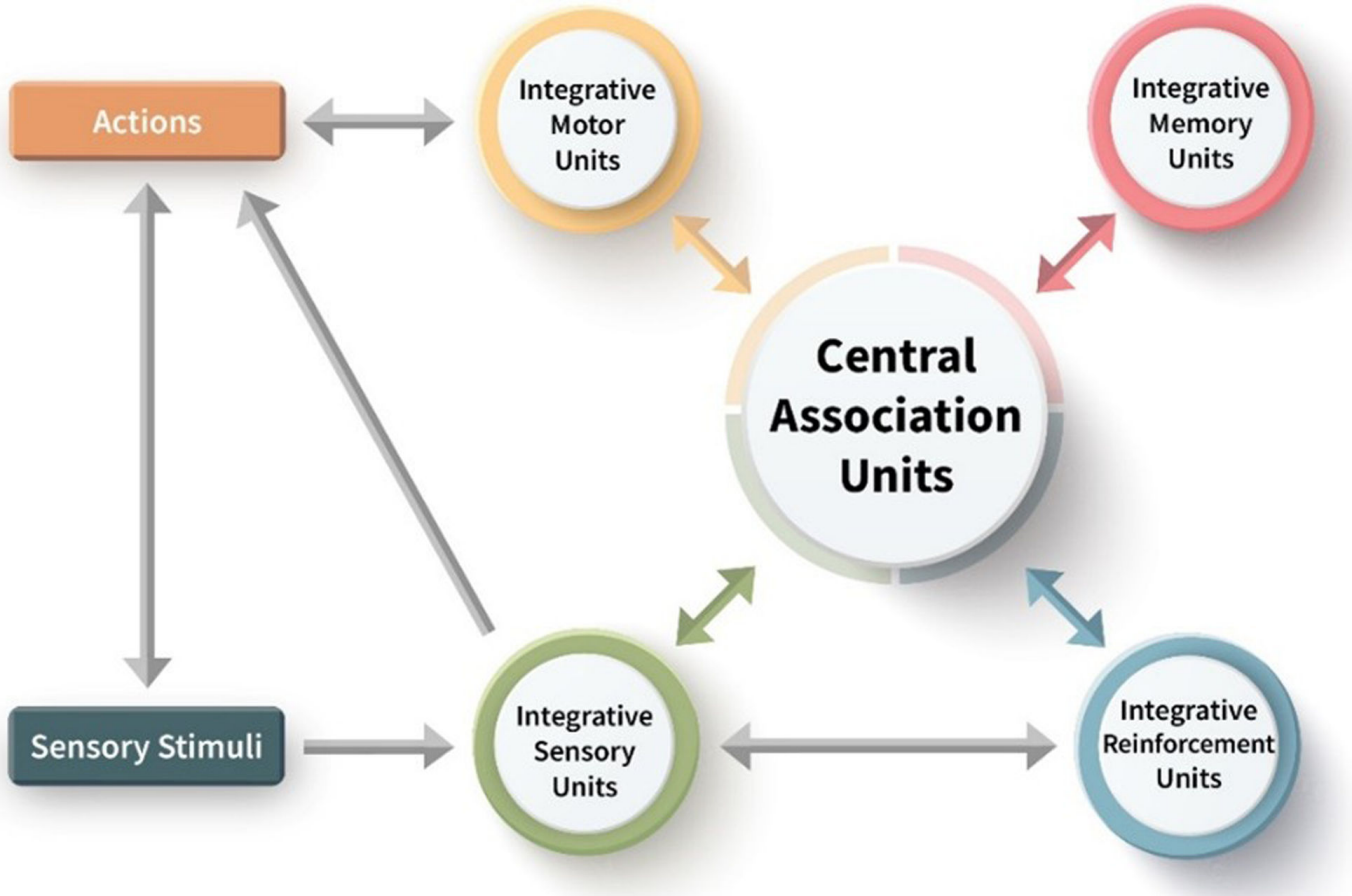
An outline of a minimal generic toy model of UAL architecture. UAL is hypothesized to depend on reciprocal connections between sensory, motor, reinforcement (value) and memory processing units at several hierarchical levels (not shown here), that come together to construct a central association unit depicted at the core of the network. We suggested that in early vertebrates the event memory unit had a dual role, acting as both a proto-declarative memory system as well as the central association unit and a global neural workspace hub [40].

What can be inferred about the enabling system that implements UAL? Which processes are necessary for its implementation? According to the UAL model, the functional cognitive architecture implementing and enabling UAL and the goal-directed behaviour that it makes possible require complex neural-cognitive organization including a hierarchy of functionally and structurally coupled sensory, motor, memory and evaluation processing units; neural selection processes that inhibit and amplify relevant effects of stimuli that are implemented by local processes at different scales (primarily amplification and inhibition between neuronal networks involving negative and positive feedback interactions); a dedicated memory subsystem that stores mapped event-representations; a dedicated evaluation subsystem that can assign valence to any compound input configuration and that enables context-sensitive prioritization; and a motor subsystem based on body mapping allowing the representation of prospective actions and a distinction between self-generated stimuli and world-generated stimuli (see figure 2 for an outline of a toy model of UAL). We assume that that there are re-entrant connections at different levels of organization among these subsystems, and that their processing effects come together within a common neural space, a central association unit. Selective stabilization, involving amplifications and inhibitions that underlie attentional processes, re-entrant connections and predictive coding are all critical to the dynamics of this system [18].

UAL requires a functional architecture that closely corresponds to the global neural workspace (GNW) architecture suggested by Dehaene & Changeux (see [41] for a review of this architecture; we pointed out to this correspondence in [18] and [40]). This functional neural architecture involves representations that are updated by current inputs and are categorized and selected on the basis of their past and present effects. We called the dynamic mapped representations underlying mental experiences categorizing sensory states, (CSSs). We suggest that CSSs require distillation processes and bowtie dynamic structures—structures that occur at intermediate layers of a multi-layered architecture and have much fewer components than the input and output layers [42,43]. Dynamic bowtie structures are observed in all signalling networks and are expected to be generated at several hierarchical levels of neural processing, with conscious awareness requiring a final information-distillation process.

It is important to emphasize that our suggestion is that *UAL is an ETM, not an ontogenetic developmental marker, of consciousness*. Although during phylogeny minimal consciousness has been constituted by UAL, the manifestation of minimal consciousness during ontogeny (such as feeling hunger) precedes behavioural manifestations of UAL that require cumulative memory. Human babies (and other young animals), are minimally conscious because they are born with active, neural-cognitive embodied architectural dynamics that evolved because of selection for UAL—an architecture that enables the integration of percepts, values and action patterns requiring working memory that lead to sentience. The behavioural-learning manifestation of discriminations, learning-dependent categorization and evaluation among composite patterns, which drove the evolution of UAL, occurs later during ontogeny because it requires the cumulative storage of memory traces. So ontogeny does not recapitulate phylogeny in this case. The activity of a UAL-able brain is sufficient for consciousness. An involvement of UAL-enabling embodied cognition also explains why some reflexes are consciously experienced—when the knee-jerk response reaches the UAL-able active brain, the reflex is subjectively experienced, although this reflex does not *require* subjective experiencing (as occurs in unfortunate individuals whose spinal cord was severed from their brain). Conversely, already consciously learned complex rules, for example the rules of chess, can become transferred to the auto-pilot mode and employed without awareness by skilled chess players.

According to our proposal, reverse engineering from UAL to the (living) system that enables it would result in the reconstruction of a system that has all the properties characterizing a minimally conscious biological organism. UAL, which is an aspect of complex cognition, points to the existence of an enabling system that is minimally conscious. We suggested that the evolution of UAL drove the evolution of an enabling living system that is minimally conscious, just as the evolution of unlimited heredity drove the evolution of minimal sustainable life. Of course, there is some gap between characterizations of consciousness that are based on behaviour, cognition and neural dynamics, and phenomenal consciousness. However, a living, autonomous, autopoietic system, which continually and cumulatively learns about the changing composite spatial and temporal aspects of the world and its own actions; which categorizes, updates and constantly revaluates the effects of its own actions and the sensory states that predict them from an internal point-of-view, and which registers the difference between self-derived and world-derived stimuli—is as close as we can get to a biological-cognitive description of a system manifesting minimal consciousness [18]. On this view, prior to the evolution of UAL the world was devoid of composite integrated patterns that could be felt and perceived—it was, to draw on an image from a well-known Zen Koan, a world in which a tree that fell in the forest made no sound.

## 3. The goals and functions of unlimited associative learning-based minimal consciousness

If one accepts that UAL is an ETM of consciousness, then the functional advantages of UAL are also some of the specific functions of consciousness. The most obvious functions are those related to the great expansion of newly learned possible associations and actions that become open to the animal:

(i) UAL enables animals to discriminate among complex patterns of percepts and activities and make decisions based on their predicted differential reinforcement (for example, which song is more exciting);
(ii) it allows pattern-completion that induces memory retrieval of scenes, events and context, so that the animal can predict future reinforcement;
(iii) the represented event-memories of past (reinforced) actions, in conjunction with current bodily states, provide the organism with clues as to what to do and motivate it to act;
(iv) it enables working memory, so that percepts and action plans can be maintained even when the stimuli inducing them are no longer present; this extends the scope of adaptive motor responses in the temporal domain;
(v) flexible, context-dependent, value reassessment and prioritization that includes distinctions between world- and self-generated stimuli is made possible;
(vi) categorization and generalization allow the organism to make educated guesses in new conditions;
(vii) it enables the construction of cumulative, functional, learned social signals and activities performed during development; and
(viii) developmental selection among multiple action possibilities based on the evaluation and prioritization of learned representations of actions (and of the precepts that predict them) requires that there is a shared global ‘workspace’ which provides the animal with a common yet updatable ‘point of view’ that ensures effective and flexible coordination of perception and action.

These adaptive learning abilities are based on predictive cues coming from the external world and the body. The recognition and processing of these cues depend on developmentally selected trial-and-error exploratory processes at both the behavioural and neural levels. It also, and critically, requires mechanisms that can distinguish between updatable self-generated and world-generated stimuli (including predictive stimuli), which are necessary for a basic sense of self and for action selection. The involvement of behavioural and neural exploration and developmental selection is also obligatory because the world and the state of the body keep changing, so sensory information and the activities and behaviours that make it useful need to be constantly updated.

Predictive coding that entails updatable priors is also mandatory because effective updating based only on bottom-up signals is not possible when the search space is too large. Predictive coding processes provide animals with variant representations or ‘hypotheses’ about the functional significance of world-cues and bodily actions, and these priors have constraining top-down effects on the outcomes of incoming stimuli [44]. This top-down control, which involves a distillation process that generates an informational bottleneck, resonates with James’ suggestion that consciousness is necessary to ‘load the dice’ of the endless activities in complex, intrinsically unstable active brains: ‘Loading the dice would mean bringing more or less constant pressure to bear in favor of its [the brain’s] performances which make for the most permanent interests of the brain’s owner; it would mean a constant inhibition of the tendencies to stray aside’ [45, volume 1, p. 140]. One can think about loading the dice as the function of consciousness, which seems to be one of the ways James thought about it, although it may be also thought of as a necessary cause; circular causation when causes lead to functions, which, in turn, alter causes, is common in biological systems and is part of consciousness dynamics.

The mental, top-down effects of activated and learned representations that were induced by sensory cues, constrain and can control some of the physiological effects of the stimuli that evoked them; they also can control, and can be controlled by, other representations. These controls enable the animal to respond to the feelings evoked by a neurally selected and activated felt representation well before or well after the stimulus that induced it became fully integrated physiologically. For example, as Derek Denton pointed out, relief of thirst is felt many minutes before the salt balance in the previously thirsty animal is restored, a fact of great survival value for a thirsty antelope at a water hole teeming with predators [46]. A delayed feeling may also occur: a badly injured conscious animal does not react to the injury with a feeling of pain, as long as a feeling of acute fear is driving its escape [47]. The mental representation of a bodily state can therefore become transiently dissociated from the physiological processing of current physiological cues, leading to adaptative responses that would be inconceivable without such dissociation. The processes allowing these partial and transient dissociations are the foundation on which adaptive strategies based on imagination, which involves selection among variant representations of virtually experienced events, had evolved [30].

What about the evolution and the functional effects of motivating desires and aversions? We argued that the UAL model can shed some light on the evolution of such motivations [31]. As Jaak Panksepp [48] insightfully suggested, the emotion he called SEEKING (an emotion entailed by spontaneous exploration) is intrinsically positively valued. The UAL model provides an evolutionary rationale for his proposal: spontaneous perceptual and motor exploratory activity of animals with UAL is positively reinforced and results in the overall sensation of pleasurable desiring, because the exercise of agency enables learning and leads to knowledge, which is adaptive. A link between the joy of perceptual agency and knowledge was already recognized by Aristotle in the first paragraph of *Metaphysics*: ‘All men by nature desire to know. An indication of this is the delight we take in our senses; for even apart from their usefulness they are loved for themselves; and above all others the sense of sight. […] The reason is that this, most of all the senses, makes us know and brings to light many differences between things’. [49, pp. 1-6]. That the exercise of sensory and motor agency is indeed inherently joyful can be clearly demonstrated after animals are liberated from sensory and motor deprivation—their joy is dramatically demonstrated [31]. An important complementary aspect was stressed by Nicholas Humphrey [26]: when the perceptual and motor exploration that is basic to living is inherently pleasurable, it motivates the animals to *care* for its survival. However, the inherent pleasure of exploration can lead, when out of control, to pathological self-stimulation, to addictions.

Experiences such as pain, anxiety and fear, feelings which come under the umbrella term ‘suffering’—are also sources of knowledge, alerting the animal to actual or potential harm to itself. Pain is unpleasant precisely because it informs the animals of its far-from homeostasis state, leading to adaptive responses protecting the individual from deprivation of agency and greater harm: individuals that do not feel pain show no self-protecting behaviour and usually suffer injuries and early death [50]. We suggested that adaptive suffering exceeds its optimal adaptive advantages, and that excessive suffering was greatest in the first conscious animals. We reasoned that reactions to partial cues of adversity which are one of the adaptations that UAL entails, commonly lead to ‘false positives’, to costly over-reactions that are overly fearful, anxious or aggressive. This ‘smoke detector principle’ as Nesse [51] called the excessive response to usually predictive signs of adversity, is unavoidable. The evolution of active forgetting partially ameliorated the felt stressful excesses of overlearning but could not altogether prevent it [18].

In spite of its inevitable costs, consciousness is advantageous and was maintained by selection in most animal lineages (it was probably lost in some lineages when the brain was evolutionarily lost or greatly simplified). Taken together, the benefits of UAL suggest that the function of UAL—and of minimal consciousness—is to form a new realm of goals based on the satisfaction of felt needs and their perceptual predictors. In this sense, consciousness, like life, is best conceptualized as a goal-oriented system, whose coupled component-processes have multiple functions that would not be possible without it [18]. This is in line with Willam James’s view of the function of consciousness: ‘Every actually existing consciousness seems to itself at any rate to be a *fighter for ends,* of which many, but for its presence, would not be ends at all’ [45, p. 141, James’s emphasis].

This general function of UAL—the formation of a new realm of goals—applies to all conscious organisms. It takes different forms in different animals, depending on their sensory, motor, memory and evaluation capacities, which are related to their evolutionary history, to the ecological niche to which they are adapted and to their individual idiosyncrasies. Birch *et al*. [52] suggested that there are five different dimensions to consciousness: perceptual richness, evaluative richness, integration at a time, integration over time, and self- consciousness, and we suggested a sixth one—the scope and control of motor agency [53]. We also argued that in lineages in which the evolution of consciousness included the addition of new levels of representation and control, as with imaginative birds and mammals, new cognitive and affective functions had evolved (e.g. causal reasoning, ‘theory’ of mind, empathy), as well as new pathologies (e.g. post-traumatic stress disorder). Imaginative consciousness involved the evolution of new integrating brain structures (the neo-cortex in mammals, and telencephalic nuclei in birds) as well as novel interactions of these structures with underlying brain layers (for discussions see [18,30]). It was the basis of human, symbol-based cognition and consciousness: new, abstract-symbolic values (the true, the beautiful, the good) drive the behaviour and the evolution of humans, who have a singular mode of being, justifying Darwin’s decision to assign to the selection that humans exert a special category—that of artificial selection.

## 4. Signal selection^4^ : the morphological, behavioural and cognitive signatures of mentally guided selection

Interactions that depend on signals exchanged between predators and their prey or between sexual partners are central to these signals’ evolution. We argue that in these, as well as in other social interaction contexts, animals capable of UAL (and therefore, according to our theory, consciously experiencing animals) respond to their world on the basis of their conscious perceptions and evaluative feelings. We further argue that these perceptions and desires have led to choices which drove the evolution of the diversity of perceptual and motor composite signalling patterns that we encounter on Earth and that they can be thought of as signatures of mental selection. In other words, without mentally guided evolution these patterns would not exist. Moreover, these patterns are indicators that there are bearers of such traits, which can be therefore identified, thus complementing other criteria for the identification of conscious beings.

### (a) Prey and predator signalling interactions

The co-evolution of predator–prey interactions is one of the most intensely studied topics in evolutionary biology and the adaptations of predators and prey are some of the best lines of evidence for the role of natural selection in evolution. Prey and predators usually have conflicting interests, with prey organisms having evolved and learned strategies of avoiding or resisting being detected and eaten, and with predators attempting to counter the prey’s strategies, leading to evolutionary arms-races. Conflict is, however, not the only way of generating co-evolutionary dynamics: plants evolved strategies for encouraging animals to feed on some of their parts, leading to competitons for attracting consumers. Our ‘prey–predator’ category therefore includes all relationships among consumers and consumed.

Here we discuss some antagonistic interactions. There are endless examples of protective and aggressive composite prey signals (informational cues), including many different types of camouflage (background matching, disruptive coloration, counter shading, masquerade, and more; for a discussion see [54]). These signals are tailored to the perceptual, cognitive and affective capacities of the predators, which were evolutionarily shaped by the predator–prey arms races. We suggest that the many evolved and learned composite signals in prey organisms including some of their behavioural defence strategies, point to the cognitive, UAL-able cognition of predators (and sometimes of prey too), which on our theory point to selections driven by perceptions and desires. Moreover, some of the mental capacities of the predators, their emotional drives and their perceptual capacities were honed by selection within this context. Here we describe only two of the numerous examples of such signal-driven evolution—two examples of visual camouflage—that would not have evolved without selection by predators endowed with an evolved perception and cognition requiring the UAL-based capacities of spatial and temporal integration, evaluative flexibility and the ability for generalization and categorization.

Our first example is of stick or leaf insects (phasmids). Stick insects’ colouring, shape and texture mimic leaves and twigs in exquisite detail, and they also exhibit motion mimicry rocking gently from side to side in harmony with the leaves they sit on when there is a breeze. One stick insect, the giant prickly stick insect (*Extatosoma tiaratum*) mimics different aspects of its ecological niche at different phases of life, with the nymphs resembling the ants in the ant-nest in which they hatch, while the adults precisely mimic the crumpled foliage on which they sit [55]. This incredible detailed camouflage would not have evolved if the ants were unable to distinguish between the many sensory aspects of the nymphs and their fellow ants, and if the potential predators of the adults, which include spiders, mantids, bats, rodents, reptiles and birds, were not able to perceive composite visual and behavioural patterns and select the less perfectly disguised individuals.

Our second example is the facultative camouflage that is displayed by coleoid cephalopods (octopus, cuttlefish, squid) that can rapidly alter their colour, shape and behaviour to mimic various aspects of their environment (including other animals). One of the most amazing is the mimic octopus, *Thaumoctopus mimicus* of Indonesia, which can mimic at least thirteen different animals including flounder fish, jellyfish, sea stars, sea snake, sponges and a crab, by changing its coloration, texture, shape and behaviour (by altering the position of its arms, inflating or flattening its mantle, and of course altering the colour and the texture of its skin). All this is done in seconds. Most of the mimicry is defensive (to avoid being recognized by its many clever predators, which include fishes, sea mammals and sea birds) but its mimicry of crabs is aggressive—it mimics the crab in order to get close to it and attack it [56]. There can be little doubt that the capacity of the octopus to produce these precisely mimicked elaborate patterns was selected by its predators’ advanced perceptual and cognitive capacities, although in this case it can be also inferred that the octopus can itself recognize the patterns that it mimics (e.g. the crab body shape and patterning), and make decisions based on its perceptions, so in this case we may infer that it, too, is conscious.

Which cognitive, perceptual and affective strategies of predators were shaped by their interactions with camouflaged prey? Galloway and colleagues [57] discuss the various evolved (and in some cases also learned) responses of predators which include increased sensitivity of the visual modality, recruitment of additional sensory modalities, adjustment of behaviours that improve search methods, ways of filtering the signal from the surrounding milieu, attention to different aspects of the context in which camouflage occurs, and causing the prey to move by flushing it out. Control of aggressive and fear emotional reactions are also likely to have been honed by selection for effective predation. In prey, fight and flight reactions, exploratory behaviour and correlated neophobic or neophilic tendences are influenced by its interactions with predators. The ability to predict actions on the basis of predictive prey-elicited cues is the basis of the ability of conscious animals to infer intentions and emotions in conscious prey.

### (b) Sexual selection by mate choice and other forms of social selection

As we noted earlier, sexual selection by mate choice was considered by Darwin to be an indication of mentality. Towards the end of the second volume of *The Descent of Man and Selection in Relation to Sex* he summarized this position: ‘He who admits the principle of sexual selection will be led to the remarkable conclusion that the cerebral system not only regulates most of the existing functions of the body but has indirectly influenced the progressive development of various bodily structures and of certain mental qualities. Courage, pugnacity, perseverance, strength and size of body, weapons of all kinds, musical organs, both vocal and instrumental, bright colours, stripes and marks and ornamental appendages, have all been indirectly gained by the one sex or the other, through the influence of love and jealousy, through the appreciation of the beautiful in sound, colour or form and through the exertion of choice; and these powers of the mind manifestly depend on the development of the cerebral system.’ [3, II, p. 402].

As Darwin documented in characteristic detail, many animals, including many insects, display complex, harmoniously organized, visual, motor, auditory, vibratory and olfactory patterns of morphology and behaviour, which often, in harmonious combinations, attract the opposite sex. The sex to which these displays are directed shows fine capacity for discriminating among subtly different displays. The complex patterns on the peacock’s tail could evolve *only* if peahens could ‘appreciate’ it: she must be able to discriminate among variant patterns and assign value to them. Similarly, the dances and songs of many bird species evolved because females discriminate among and prefer complex dances and songs, sometimes visiting sequentially several males in different locations or scrutinizing a multimale display. In all such cases, including those of the impressive architectural constructions of male bower birds, the composite social signs of mate quality and attractiveness evolved because females can perceive, compare and evaluate them. A spectacular example of multi-modal complex mating display in an invertebrate, is that of the Australian peacock spider, *Maratus volans*, a jumping spider whose abdomen flaps are adorned with a blue, orange and gold splendid pattern. When approaching a female the colourful flaps are displayed, the male raises its third pair of legs, waves them (thus also showing a brush of black hairs and white tips) and sways from side to side while vibrating the flaps in front of the female. Males who do not conform to the female’s high standards and do not manage to excite her through a satisfactory multi-modal display are selected against: they are in danger of being eaten by the female [58].

The evolution of the visual and olfactory patterns of flowers depends on the ability of pollinating insects and birds to discriminate among their visual, tactile and olfactory patterns, and thus inadvertently pollinate them. Flowers pollinated exclusively by wind are colourless and have no smell and taste [16]. Darwin devoted a major part on his book on the fertilization of orchids [59] to this kind of selection, admiringly writing about the amazing mimicry of the female bee by bee orchids, which attract male bees. Only discerning pollinators like these male bees, with an ability to discriminate between more and less female-bee-like orchid flowers, can lead to the evolution of such precise multi-modal mimicry. It is the evaluator of the sign who needs to be conscious (the sender of the sign may or may not be conscious)—in this case it is the evaluation of signs by conscious receivers and their consequent decisions, that has led to complex pattens of colour and smell in non-conscious flowers. If an interpreter (the male bee for example) cannot perceive, compare, categorize and evaluate different composite and salient patterns it cannot choose between them and cannot drive their evolution.

The co-evolutionary dynamics of sexual selection by mate choice may also lead to the selection of mental faculties in the sender, something that Darwin was well aware of: ‘If female birds had been incapable of appreciating the beautiful colours, the ornaments, and voices of their male partners, all the labour and anxiety exhibited by them displaying their charms before the females would have been thrown away; and this it is impossible to admit.’ [3, I, pp. 63−64]. Both female birds’ appreciation and male birds’ anxiety and desire had evolved in the context of on-going sexual inter-mate selection, allowing both sexes to infer, on the basis of predictive cues, the emotional state and the intentions of their partner.

Our suggestion regarding the functions of consciousness, which are based on our UAL model, support Darwin’s position, but do not commit us to any particular theory regarding the selection regime driving the co-evolution of preferences and display signals. Such co-evolution could be the result of advertisement of ‘good genes’ as suggested by Zahavi & Zahavi [60], or of the runaway selection suggested by Fisher [61], or both. What we argue is that whatever the selection regime driving the co-evolution of displays and preferences, the choosing partner has to be able to perceive, compare and emotionally evaluate composite signals, and that it is the capacity to evaluate composite signals that had led to the evolution of these display signals in the sending partner, and the motivation to preferentially respond to them in the receiver. In some cases of mate choice, social, rather than individual learning is involved: in some fish and bird species naive females copy the mate choice of an experienced female (e.g. [62]). In these cases, the copying females must retain over some time the characteristics of the chosen male, a cognitive process that requires UAL cognition.

Though common and found in vertebrates and in invertebrates such as crustaceans, spiders and insects, selection by mate choice is not found everywhere. Darwin found no evidence for sexual selection by mate preference in protists, coelenterates, echinoderms and annelids, and attributed this absence to the ‘lower mental powers’ and the ‘imperfect senses’ of these animals. However, although he thought that *positive evidence* of sexual selection points to complex mentality and what he called ‘a taste for the beautiful’, he did not regard the *absence* of sexual selection as definite evidence for a lack of mental powers. He found no evidence for sexual selection in cephalopods, whose high intelligence, which he associated with mentality, he recognized. Sexual selection based on mate choice of complex displays that require flexible discrimination ability as well as working memory and categorization, is one positive indicator for UAL (and of minimal consciousness). As we argued, there are other indicators, which are even more common—composite signals related to the attainment of food, especially predator–prey interactions.

There are also additional interactions where selection for signals had occurred. Sexual selection by mate choice is only one form of social signal selection. Selection for offspring recognition, selection for the recognition of individual group members and the relationships among them are additional examples of social selection. The huge and sometimes elaborately marked gaping mouths of nestling birds are an example of selection for signals that elicit offspring recognition and care in parents. The most remarkably ornamented are those of the mouths of the nestlings of grass finch species that have a luminous papillae lining the gape and an elaborate pattern of spots and bars on their palate. These markings vary among grass finch species, and their evolution may have been driven, in addition to sibling competition for parental care, by their parasites, indigo birds, who parasitize the nest and whose nestlings have evolved similar markings as those of the grass finch species they parasitize [63]. What we want to highlight here, as in the case of predator–prey interactions, is not the specific selection regimes that drove these evolutionary arms-races, but the simple inference that the evolution of such patterns would not have been possible had grass finch parents been unable to discern, compare and evaluate differences among mouth patterns of their gaping young.

Social selection involving composite cues is also seen in social insects. In some bees, certain wasps and ants, the workers kill all but one of several queens [64] . The workers identify the doomed queens either by the smell of the queen herself, or, in the case of stingless bees, on the basis of the smell of lazy and unproductive diploid males that she produces because of mating with the wrong partner [65]. Wax wasps which live in colonies have individually variable face features which seem to have evolved to enable individual recognition of colony members [66]. These and the numerous other cases of complex and composite, often multimodal, social signals, point to an ability for discrimination and decision-making that depends on UAL-based cognition. Many social behaviours and affects, such as the motivation to explore through play, the ability to infer what the emotional states of interactants are, the scope of emotional contagion and social-emotional preferences, are evolutionarily incomprehensible without assuming an evolvable UAL-able, conscious mind. Although we cannot at present estimate how many compositional steps in the brains of signal-receivers are required to enable the assembly, synthesis and analysis of composite spatial and temporal selectable patterns, we believe that assembly theory [13] offers a promising research agenda for providing quantitative estimates of the selection steps required for signal-detection.

Many selection regimes may have driven predator–prey, sexual and other social interactions and signalling, but our approach suggests that learning probably initiated the co-evolution of preferences and signals. On our theory, since conscious animals are capable of UAL, they have surely employed this highly adaptive learning capacity to locate prey and discriminate among mates and social partners during their lifetime. Their learning led to partial genetic accommodation of their preferences and of related supporting cognitive and affective characteristics [67,68], which are indicative of mentally guided selection and of conscious signal-interpreters.

## 5. The Cambrian explosion

UAL is a testable set of learning capacities and its distribution in the living world can be determined. Our survey of the learning literature had revealed that UAL has been found in all tested vertebrates, in some arthropods and in the coleoid cephalopod molluscs (octopus, cuttlefish and squid). Since the brain structures that support UAL in these groups were identified and since fossilized brains of members of these groups were found, it is also possible to determine when, and infer how many times, UAL evolved. The fossil record suggests that UAL-enabling brain structures first appeared in vertebrated and arthropods during the Cambrian era (approx. 540–485 Ma), a historic evolutionary big bang when almost all currently existing animal phyla originated and diversified, called the ‘Cambrian explosion’. The coleoid cephalopod molluscs appeared in the fossil record around 250 million years later, in response to the strong selection pressure imposed on them by fish predators [69]. In these three phyla UAL evolved from simpler forms of associative learning (non-associative learning and limited associative learning, which do not entail consciousness) in the context of biotic antagonistic and cooperative interactions during and after the early Cambrian [18]. It therefore seems that UAL cognition that entails minimal consciousness evolved independently twice (if arthropods and vertebrates had a common UAL-able ancestors) or three times (if UAL evolved independently in the three groups) and was probably lost several times when the brain was lost in some lineages in these phyla (see [18,27,70]).

We suggested that the momentous effects of associative learning, and particularly of UAL, were one of the factors that drove the Cambrian explosion. Once UAL and minimal consciousness evolved, selection became dependent on the conscious perceptions and choices that drove the evolution of complex cognitive patterns of action and motivating affect. The many advantages of associative learning and especially of UAL led to a quantal leap in adaptability: associatively learning animals were more effective predators, more evasive prey, more discriminating mates, more discerning cooperators than organisms that were unable to learn in this way. They exerted, through natural, sexual and other forms of social selection, heavy pressure on conspecifics and interacting species, leading to co-evolutionary races, to the rapid evolution of adaptations and counter-adaptations and the corresponding, rich patterns of signalling, such as those described in the previous section. The mentally guided selection that started during the Cambrian remained a major factor in evolution ever since, leading to new forms of adaptive diversity, including the imaginative consciousness of some animals and the symbol-based introspective consciousness of humans.

## 6. Discussion: mental selection and its global effects on the web of life

We suggested that *mental selection*, selection guided by mental states, which is the product of the evolution of complex UAL-enabling cognition in animals, should be considered a distinct category of natural and developmental selection. Consciousness is a mode of being, which, once in place, has opened up new possibilities of interaction and of selection. The specific goals and functions of conscious agents led to evolutionary patterns that would be impossible without consciousness, and which make it possible to identify the bearers of consciousness, which drive the evolution of such signals. Some forms of natural and sexual/social selection are based on conscious (perceived, felt) choices and this makes a profound difference to adaptive evolution: selection that is based on consciously experienced perceptions, voluntary actions, evaluative feelings (and in imaginative animals, also on virtual experiences), enables animals to choose composite spatial and temporal patterns of predictive percepts and actions that are not available to non-conscious organisms. Mentally guided selection therefore has evolutionary effects that are not conceivable without consciousness. When only the evaluator of a signal is conscious, complex signalling patterns can be selected and evolve in non-conscious signallers, driving the evolution of motivational features in their conscious evaluators. When both evaluator and signaller are conscious there will be, in addition to the co-evolution of signalling morphologies and behaviours, also co-evolution of the relevant dimensions of consciousness in both social partners. Selection guided by mental states has been a necessary condition for the later emergence of rationality and artificial selection by rational human agents.

As we stressed, it is not just conscious animals that evolve new types of composite adaptations, but all the organisms, conscious and non-conscious, that interact with them. It is difficult to think about organisms, on land, in the sea and in the air, that do not interact with conscious vertebrates and arthropods (in the sea, some organisms also interact with conscious coleoid cephalopods). The web of interactions in the living world is extensive and tangled and consciousness-based interactions among organisms, began to reverberate through it as soon as consciousness first arose in the ancient seas of the Cambrian. The biotic interactions that are related to the need for food, safety, sex and additional social relations, led to adaptations that would not be possible without the involvement of UAL-able, consciously selecting agents. If beings on other planets followed a pattern of evolution broadly similar to that which we observe on Earth, the existence of complex, discriminable, sensory communication signs may be considered as signatures of mental selection of past or present conscious life on these planets.

Our claim is based on our approach to consciousness, according to which, the kind of cognition that enables UAL entails minimal phenomenal consciousness. This is not a generally accepted theory even among biologists and philosophers who believe that consciousness is an evolved mode of being that emerged in some neural animals. Some scholars, like Walter Veit [25] and Louis Irwin [71], would argue that UAL is too demanding, that an evaluative capacity based on feelings that entails a sense of self is sufficient for consciousness. We argued that such evaluative capacity requires the whole UAL-enabling cognitive package and the functions of felt evaluations depend on the coupling of this representational capacity with other cognitive capacities. Another objection to our claim is that we are not demanding enough: Nicholas Humphrey would argue that UAL may be a marker of what he calls ‘cognitive consciousness’, which involves access to a global workspace and is expressed as intelligent (UAL-able) cognition but does not involve phenomenal consciousness. In Humphrey’s view, phenomenal consciousness, unlike cognitive consciousness, is more specialized and later evolved. It can be detected when conscious animals (some birds and mammals according to Humphrey) engage in what he calls ‘natural psychology’, which includes understanding others’ emotions and beliefs, imaginative play, and episodic-like memory [28]. The functions of consciousness according to this view would therefore overlap the functions enabled by what we called imaginative consciousness, rather than the more general functions that are required by a UAL-enabling cognitive architecture. We disagree with Humphrey because as argued in §2, UAL *entails* the sense of self that is central to his theory as well as all the other generally agreed-upon attributes of consciousness that we listed (the more elaborate sense of self that requires natural psychology came later, according to our view). Although, like Humphrey, we believe that the GNW theory describes the neural dynamics of consciousness, the original human-based GNW model can be adapted to brain dynamics in of basal vertebrates (primitive fishes) and possibly also to arthropods and cephalopods. (In fishes, the GNW dynamics, and by implication minimal consciousness, can be shown to be instantiated by the hippocampal homologue [40]).

Which evolutionary theory of consciousness is most likely will be decided by testing the predictions of the different theories of consciousness. We predict that experimental protocols such as backward masking that selectively switch off conscious perception in humans, leaving unconscious perception in place, will selectively switch off UAL in humans and other UAL-able animals, while leaving more limited forms of learning in place; that blind-sighted individuals will be unable to perform UAL tasks on stimuli presented in the blind region of the sensory field, but may be capable of more limited forms of associative learning presented on the blind side; that neural signatures of subjective experience will be correlated with neural signatures of UAL, and that comparative studies in humans and other UAL-able species as they awake from anaesthesia are expected to reveal activity patterns in the brain regions implementing UAL (additional predictions of the UAL theory are presented in Birch *et al*. [37], a more exhaustive list is presented in [33]).

The argument advanced in this paper is that without the cognitive functional capacities enabled by UAL, which entail consciousness, and without the mental selection that consciousness makes possible, the patterns observed in the living world would not have the richness and intricacy we observe. The attempt to inform (and reform) the philosophy of mind by taking an evolutionary approach to the mind is an ongoing and arduous endeavour, and we expect that the attempt to make a difference to theorizing about evolution by taking on board consciousness studies and the naturalistic philosophy of mind will be equally difficult. However, we need both.

## Data Availability

This article has no additional data.

## Footnotes

^1^
Since the terms goal, value, function and adaptation are sometimes inconsistently characterized in the literature, here is how we use them in this paper: we attribute a *goal* to a system when it has a value that guides its behaviour; *value* is intrinsic reinforcement that guides goal attainment through a process of selection (examples of values are survival and reproductive success; fulfilment of phenomenally felt needs; and in humans—abstraction such as truth, beauty, justice and freedom). *Function* is attributed to a trait (structure, process, relation) that systemically contributes to the goal-directed behaviour of the encompassing system, so *adaptation* is a naturally, mentally or rationally selected functional effect.

^2^
Tinbergen did not consider mental causation as a cause because he did not think that we have the tools of studying it, but it seems that he did not think that the framework he suggested was hampered by the inability to study mental causes; nor did he suggest that mental causation has mental-specific functions.

^3^
In the current paper, we do not discuss the evolution of UAL from simpler forms of cognition. The transition to UAL (and minimal consciousness) from more basic forms of cognition, as well as a general definition of cognition are discussed in [34].

^4^
The biosemioticians rich framework, which focuses on signalling, does not include the role of non-human animal consciousness in the analysis of signals’ evolution. For discussions of the evolution of animal consciousness from a biosemiotic perspective see responses to [31] target article, which are referred to in Jablonka & Ginsburg’s [32] response to the commentators.
